# Reconstructing SARS-CoV-2 infection dynamics through the phylogenetic inference of unsampled sources of infection

**DOI:** 10.1371/journal.pone.0261422

**Published:** 2021-12-15

**Authors:** Deshan Perera, Ben Perks, Michael Potemkin, Andy Liu, Paul M. K. Gordon, M. John Gill, Quan Long, Guido van Marle

**Affiliations:** 1 Department of Medicine, Cumming School of Medicine, University of Calgary and Alberta Health Services, Calgary, AB, Canada; 2 International Baccalaureate Diploma program, Sir Winston Churchill High School, Calgary, AB, Canada; 3 Department of Microbiology, Immunology, and Infectious Diseases, Cumming School of Medicine, University of Calgary, Calgary, AB, Canada; 4 Department of Biochemistry & Molecular Biology, Alberta Children’s Hospital Research Institute, University of Calgary, Calgary, AB, Canada; 5 Department of Medical Genetics, and Mathematics & Statistics, Alberta Children’s Hospital Research Institute, O’Brien Institute for Public Health, University of Calgary, Calgary, AB, Canada; University of Helsinki: Helsingin Yliopisto, FINLAND

## Abstract

The COVID-19 pandemic has illustrated the importance of infection tracking. The role of asymptomatic, undiagnosed individuals in driving infections within this pandemic has become increasingly evident. Modern phylogenetic tools that take into account asymptomatic or undiagnosed individuals can help guide public health responses. We finetuned established phylogenetic pipelines using published SARS-CoV-2 genomic data to examine reasonable estimate transmission networks with the inference of unsampled infection sources. The system utilised Bayesian phylogenetics and TransPhylo to capture the evolutionary and infection dynamics of SARS-CoV-2. Our analyses gave insight into the transmissions within a population including unsampled sources of infection and the results aligned with epidemiological observations. We were able to observe the effects of preventive measures in Canada’s “Atlantic bubble” and in populations such as New York State. The tools also inferred the cross-species disease transmission of SARS-CoV-2 transmission from humans to lions and tigers in New York City’s Bronx Zoo. These phylogenetic tools offer a powerful approach in response to both the COVID-19 and other emerging infectious disease outbreaks.

## Introduction

Public health programs are often challenged by outbreaks caused by novel or re-emerging pathogens. Appropriate and immediate public health interventions are then required to prevent an uncontrolled epidemic or global pandemic from developing [1, 2]. In the 21^st^ century alone, the world has experienced outbreaks of the Ebola virus, Zika virus, new strains of Influenza viruses, and the present COVID-19 pandemic caused by SARS-CoV-2 [1, 3, 4].

Whole-genome viral sequencing when applied in genomic epidemiology, plays an increasingly important role in investigating many infectious disease outbreaks [5]. The COVID-19 pandemic reinforces the potential importance of these newer approaches. The primary goal of any epidemiologic investigation is the mitigation and termination of disease spread. Analysis at the nucleotide level using state-of-the-art sequencing technologies can be used for the characterisation of pathogens and their transmission patterns [2, 6]. The viral genomes of in particular rapidly mutating RNA viruses, generate sufficient genetic diversity for the inference of the pathogen’s transmission. Therefore, genomic epidemiology is becoming a feasible and useful tool to infer viral epidemiological dynamics, solely through the use of viral genomic data. Such data is increasingly available from diagnostic testing across the entire timeline of an epidemic [1].

Complete and accurate inference of infection transmission networks can enable effective protocols to be implemented to mitigate disease spread [1]. However, in practice, such an approach is usually hampered by the inability to completely sample an entire population [7]. Unsampled sources of infection play a significant role in infectious disease transmission leading to the rise of unexpected clusters of infection. They also impede with estimating the burden of infection in a population [7, 8].

Many of the challenges in the COVID-19 pandemic relate to the difficulties with tracking virus spread within a community [9]. Some SARS-CoV-2 infected individuals may be asymptomatic or pre-symptomatic, while being infectious to others. This leads to the presence of unknown or unsampled sources of infection within a community [10]. These sources could explain in part high infection rates, atypical clusters and even unaccounted for cross-species transmissions as seen in this pandemic [8, 11]. Systemic ways of inferring SARS-CoV-2 transmission networks is crucial to correctly estimate the number of asymptomatic or undiagnosed sources, with the ultimate goal of reducing virus transmission.

A multitude of approaches aimed at inferring inter-host viral transmission using within-host evolutionary dynamics exist. These include the work conducted by Didelot et al., Stapleton et al., and Xu et al. [7, 12, 13]. We have previously used and extended these well-established methods to estimating HIV transmission and unknown sources of infection in a population [14]. Moreover, these tools have been applied to infer the transmission of SARS-CoV-2 [15–17]. In this study, we fine-tuned these tools and extended the analyses to infer both transmission networks and infer the presence of unsampled sources of infection. Using publicly available SARS-CoV-2 genomic data, we were able to get insights in sampling approaches, the potential success of physical distancing, and show how certain infected clusters are connected through the inference of unknown sources of infection. The pipeline was also able to infer the SARS CoV-2 infection of the lions and tigers at the Bronx Zoo in New York City, USA whose infection raised much interest [18].

Our analyses demonstrate the ability of phylogenetics using limited data sets to infer the presence and of unsampled sources contributing to viral spread. This ability may assist in answering questions regarding both the direction of transmission and how certain infected populations are connected. The latter would be beneficial for better modeling of outbreaks and assist with building focused public health responses.

## Methods

### Overview of the phylogenetic pipeline

**Fig 1:**
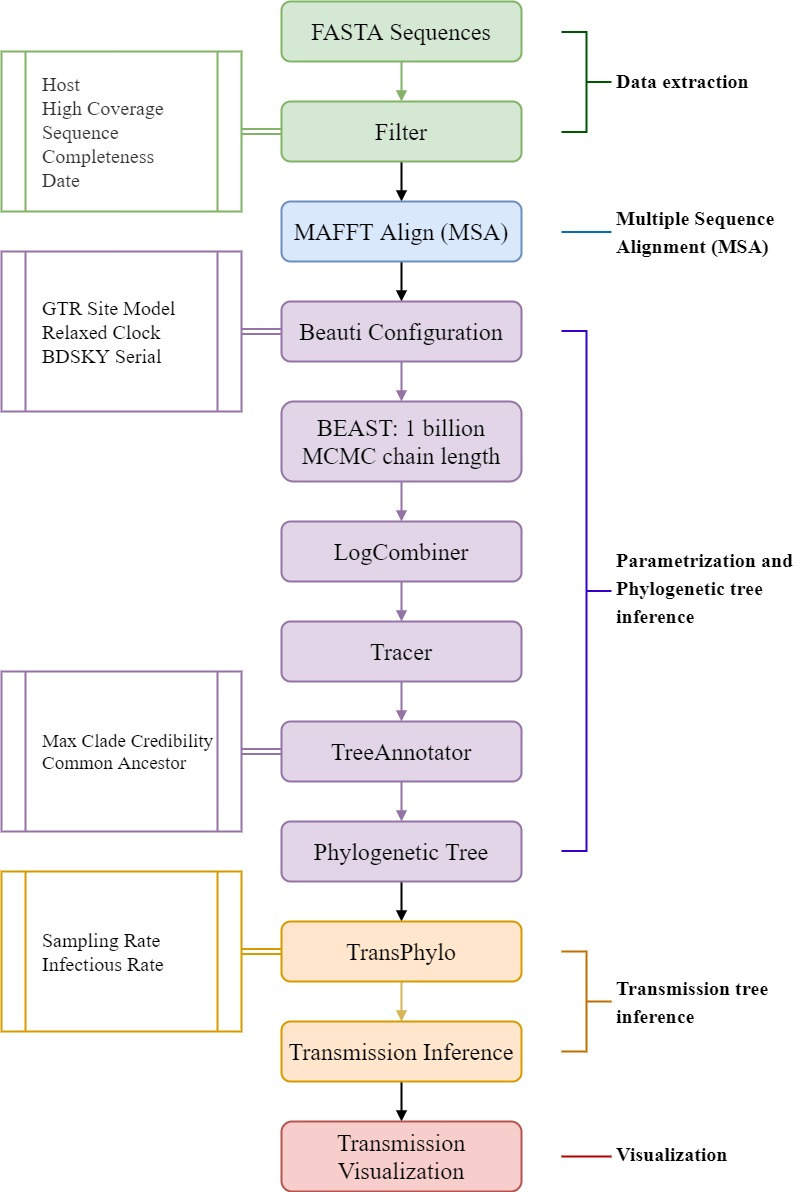
Detailed overview of the proposed five-step pipeline. The parameters that were optimised to fit the dynamics of the SARS-CoV-2 virus are depicted on the left.

The phylogenetic pipeline and approach used is shown in Fig 1. The process consisted of five steps: 1. Data extraction, 2. Multiple Sequence Alignment (MSA), 3. Parameterization and phylogenetic inference 4. Transmission tree generation and 5. Data visualization.

Through multiple testing and iterations, and comparison to publicly available epidemiological data, this multistep process was fine-tuned specifically for SARS-CoV-2. The generated transmission networks of SARS-CoV-2 were again compared with preexisting epidemiological data via literature reviews, to assess the validity of the inference of unsampled sources of infection. All the codes for the pipeline with examples can be found at https://github.com/theLongLab/TransCOVID and described in more detail below.

### Description of the Canadian, Russian, and New York State data sets

The selected data source was GISAID (https://www.gisaid.org/) [19]. We initially evaluated the data from Canada, Germany, France, the African continent, South Korea, Russia, and New York State for testing the methodology. We were able to finetune the pipeline so that it was both robust in its application and inference capabilities (see below). For our final analysis we focused on Canada, Russia, and New York State (S1 Table). Each data set offered different types of challenges for appropriate parameter settings for each use case, and were good scenarios to test the pipeline and its inferences.

The Canadian dataset was concise. The sampling rate at the time of data collection (January of 2020 to July of 2020) was adequate with just over 4 million tests being performed (with a weekly average of over 55 000 tests at 0.8% positivity), of which about 116 000 had tested positive. The public health interventions appeared to control the infection as there was a clear drop in the average number of weekly reported cases from over 2000 cases to well below 500 by early July [20]. The selected dataset spanned across several provinces, but the majority of data available at the time was for 3 provinces namely, Quebec, Ontario, and British Columbia. The entire Canadian dataset for the COVID-19 pandemic in that time interval was relatively well documented allowing us to validate our results of inferred unsampled cases [21].

The dataset collected in Russia (March 2020 to September 2020) was similar to the above dataset but appeared to be sampled at a lesser rate, suggesting a larger number of unsampled sources to be remaining in the population. By late September, Russia, was performing a weekly average of 90 000 tests, with over 2.2% being positive at over 7 200 cases. However, it was reported that about 8 000 new cases were reported. Additionally, Russia observed a spike in their COVID-19 cases post September 25^th^ probably associated with difficulties rolling out comprehensive mitigation protocols and contact tracing [22]. Based on the epidemiological data available in the GISAID database, the pipeline needed to be optimized to correctly infer the directions of transmission resulting in the accurate mapping of infected population clusters under these circumstances [19].

The New York State data represented a different set of factors and assumptions to address. During the study period (February 2020 to September 2020), New York state was considered a COVID-19 epicenter with the second-highest cases numbers of COVID-19 in the USA in March 2020. It also had a large population of people traveling into the region and physical distancing practices had not been strictly implemented [23, 24]. Similar to the Russian dataset the New York State had a lower sampling rate in comparison to its rate of disease incidence [25]. Similar to Russian dataset New York State too showed a marked increases in the number of cases from mid-June to September reporting over 1 000 new dases daily [22, 26]. These dramatic spikes in disease incidence and the accompanying lack of sampling could result in the presence of potential hidden super spreader events to be present in the population data set. Therefore, this would be a challenging data set for the phylogenetic pipeline. As further test, we included the SARS-CoV-2 sequences obtained from the lions and tigers at the Bronx Zoo, New York. The sequences from the zookeepers were not present in GISAID and therefore were not included in the dataset to ensure the continuity of quality control standards of the data. We expected the pipeline to be able to infer the cross-species transmission despite this, enabling us to get a deeper insight on how well the pipeline could infer viral transmission chains.

### Viral sequence collection and quality control

Both FASTA sequence data and the corresponding epidemiological data were downloaded from GISAID (https://www.gisaid.org/) [19]. The accession numbers of the sequences used are described in the S1 Table. We confirmed that the selected data had complete sequences with high sequence coverage. According to the inhouse filter present in GISAID complete sequences comprised of genomes with lengths greater than 29,000 nucleotides, high coverage were sequences with less than one percent of undefined bases, and low coverage were those with greater than five percent of undefined bases. Using a bespoke filtering algorithm, sequences with complete collection dates (with day, month and year) and locations were selected ensuring compatibility for the generation of timed phylogenetic trees in the subsequent steps.

### Multiple Sequence Alignment (MSA)

MSA was performed using the Multiple Alignment using Fast Fourier Transform (MAFFT) algorithm [27]. MAFTT was chosen due to its robustness in handling large datasets as well as its speed and efficiency, which comes with minimal cost to the accuracy of the alignment [28–30].

### Phylogenetic parameter selection

Phylogenetic reconstruction was performed through Bayesian time-trees employing BEAST 2.6.2 [31]. The analysis was conducted with activated tip dates, a Generalized Time Reversible (GTR) site model with a gamma category count of 4, a relaxed clock model, and a Birth-Death Skyline Serial model as the prior. Parameter selection was influenced by our previous work on HIV transmission and other publications using these tools for SARS-CoV-2 (see below) [14–16, 32].

### Phylogenetic tree generation

The total Markov chain Monte Carlo (MCMC) chain length stood at 1 billion which was performed as 10 separate runs each of chain length 10^7^. The individual runs were then merged using LogCombiner 2.6.2 and the validity of the MCMC run was evaluated using Tracer 1.7.1 by reference to the Estimates Sample Size (ESS) of each inferred parameter [33]. It was ensured that the ESS was greater than 200 for each parameter. Subsequently, the phylogenetic tree was extracted through TreeAnnotator 2.6.3 using common ancestor node heights and a target tree type of maximum clade credibility.

### Transmission tree inference

Transmission tree inference was conducted using the Bayesian program TransPhylo; a dedicated software designed to reconstruct transmission networks from timed phylogenetic data [7]. TransPhylo is particularly well suited and has been used for different COVID-19 dataset analyses [32]. TransPhylo enables the inference of transmission trees for an ongoing pandemic complete with unsampled sources of infection and the date of infection. The identification of unsampled sources was particularly important in identifying how certain clusters were connected and understanding the direction of viral transmission. TransPhylo was executed with parameters that represented viral generation times within 1 to 14 days with a median of 5.5 days and sampling times of 2 to 14 days with a median of 7 days. It should be noted that these parameters had to be varied, usually within these boundaries based on the geographical region of focus (See section “Parameter Optimization of the Pipeline” below).

### Visualization of the viral transmission networks

Transmission data tables obtained using TransPhylo were visualized using Gephi 0.9.2 an established network analysis software [34]. Gephi’s built-in clustering algorithms Force Atlas 2 [35] and Yifan Hu [36] were used to identify population clusters in the transmission network as well as visualize the data in a comprehensible manner.

### Parameter optimization of the pipeline

Using the five-step process of the pipeline, we examined the available data for Canada, Russia, and New York State (GISAID accession numbers described in the S1 Table**)**. The MCMC runs conducted in both the inferences of the phylogenetic tree by BEAST and the transmission tree by TransPhylo were statistically validated by examining their trace diagrams.

We ensured that all BEAST-generated results had Estimated Sample Sizes (ESS) above 200. The detailed configuration of the BEAST2 and TransPhylo setups is depicted in Tables 1 and 2 respectively. Through multiple runs and tests, we settled upon these parameters based on the statistical validity of the MCMC runs by obtaining sound values for Estimated Sample Size (ESS) and ensuring the MCMC chains reach convergence for the different values.

**Table 1:** Parameter configuration for BEAST2 phylogenetic tree inference.

Criteria	Parameter		Validation/ comments
Site model	Model	Gamma Site model	Gamma category count was set to five to enable variation for each site in the substitution model. Parameters of GTR were kept constant
	Substitution Rate	1.0	
	Gamma Category Count	5	
	Shape	1.0	
	Proportion variant	0.0	
	Subst Model	GTR	
Clock model	Relaxed Clock Log-Normal		Default parameters
Priors	Birth Death Skyline Serial Model		Default parameters
MCMC	Chain Length	1 000 000 000	Separated into 10 × 10^7^

**Table 2:** Parameters configuration for TransPhylo transmission tree inference.

Criteria	Parameter		Validation
Generation time distribution	Shape	1	Generation time was made variable so that it can be estimated using the MCMC run.
	Scale	0.01917	
	Unfixed		
Sampling time distribution	Shape	1	Sampling time was made variable so that it can be estimated using the MCMC run.
	Scale	0.03836	
	Unfixed		
MCMC	200 000		

Once the results were obtained the separate MCMC runs were combined using LogCombiner and the trace diagram was viewed using Tracer. A sample of the Tracer overview diagram for New York is shown in S1 Fig.

As shown in Table 2 TransPhylo was executed with parameters that represented the distribution of the generation times within 1 to 14 days with a median of 5.5 days. Similarly, sampling times were represented by a distribution of 2 to 14 days with a median of 7 days.

These parameters were made variable in the MCMC chain as these distributions varied based on the geographical region under study. These distributions take the form of gamma distributions, this is a common assumption made in epidemiology to explain both sampling and infection dynamics in a population and is adopted by TransPhylo [7, 32]. However, we also tested this assumption by checking whether the data does in fact fit to a gamma distribution using the R package fitdistrplus [37].

Finally, along with the transmission network, TransPhylo provides a trace diagram of its MCMC diagram (S2 Fig**)**. This to ensure that the estimated parameter values have reached convergence.

### Statistical validation of the pipeline

We adopted a guided “trial-and-error” approach to validate our pipeline and ensure its scalability. This approach consisted of testing parameters guided by our knowledge from our previous work on HIV [14], and through multiple passes of refinement of parameters obtained through model testing the COVID-19 data using BEAST and those reported in the literature [16, 38]. This allowed us to fine tune the parameters for the BEAST and TransPhylo models to the COVID-19 data. We first experimented with small datasets ranging from a few hundred sequences to ensure that the MCMC chains provided robust results.

As mentioned, the selection of MAFFT (Multiple Alignment Fast Fourier Transform) was done based on its reliability as an accurate and fast tool for sequence alignments involving large datasets such as ours and assumes a common ancestor [27–29].

Our first adjustments to the phylogenetic inference were made to the GTR site model as the base substitution rate inferences were not sufficiently robust as they produced ESS values less than 200. We were able to resolve this issue by increasing the Gamma category count to 5.

Secondly on increasing our data sizes to over a few thousand sequences, with more complex problems (such as sequences from multiple species, sequences from regions with limited implementation of physical distancing) we observed another decline in ESS values. We were able to mitigate this by increasing the chain length. This was incorporated by breaking the analysis into 10 identical runs with each MCMC chain length spanning to 10^7^ and combining the results.

Due to the dynamic nature of the sampling rates and virus generation times based on the region under study in the transmission inference by TransPhylo, we kept the parameters unfixed. We also increased the chain length until consistency for the inferred values was reached.

## Results

Using the richness of the GISAID (https://www.gisaid.org/) [19] data, we tested and optimized the phylogenetic pipeline for the analysis of unsampled/undiagnosed sources of infection in the context of the COVID-19 pandemic. Through consecutive tests, we examined the pipeline in terms of its statistical soundness in addition to authenticating its inferences through fact-checking with publicly available epidemiological and other data.

As described in the methods, for the validation process, we focused our analyses to the data for Canada, Russia, and New York State as there was more additional data available apart from sequencing data, such as for instance testing numbers. We felt that this information would allow for a more detailed analysis with regard to inferring unknown sources of infection in these regions over the other.

The MCMC runs conducted in both the inference of the phylogenetic tree by BEAST and the transmission tree by TransPhylo were statistically validated by examining their trace diagrams. We ensured that all BEAST-generated results had Estimated Sample Sizes (ESS) above 200.

Upon close inspection, certain parameter values were not entirely consistent with the available literature. Depending on the size of the data sets we found reproductive rates from 0.2 to 0.3 to values between 0.4 and 0.8. Several outliers giving a much higher value were also observed, ranging anywhere from 1.0 to 2.5. It was determined that the majority of these values were inaccurately small compared to literature [39, 40]. Billah et al. determined by analyzing 42 studies on three different databases, that the average worldwide reproductive rate ranged from 2.39 to 3.44 [39]. Other studies for Russia, and the United States, but also those including data from other areas such as France, Germany, China, South Korea, found reproductive rates ranging from 0.26 to as high as 6.69, depending on the extent of the pandemic in those countries. [39–41]. The most likely reason for this is the short time span and the relatively small number of samples. However, we observed that down the line these errors had minimal to no effect when inferring transmission patterns and inferring infection dates, through TransPhylo. It could be stated that this was mainly due to the fact that the viruses’ reproductive rate is dependent on the region under study due to mitigation strategies and other regional factors. Since our analysis were region based, we were able to account for this for the analyses for Canada, Russia and New York State.

### The analysis of the spread of SARS-CoV-2 in Canada

**Fig 2:**
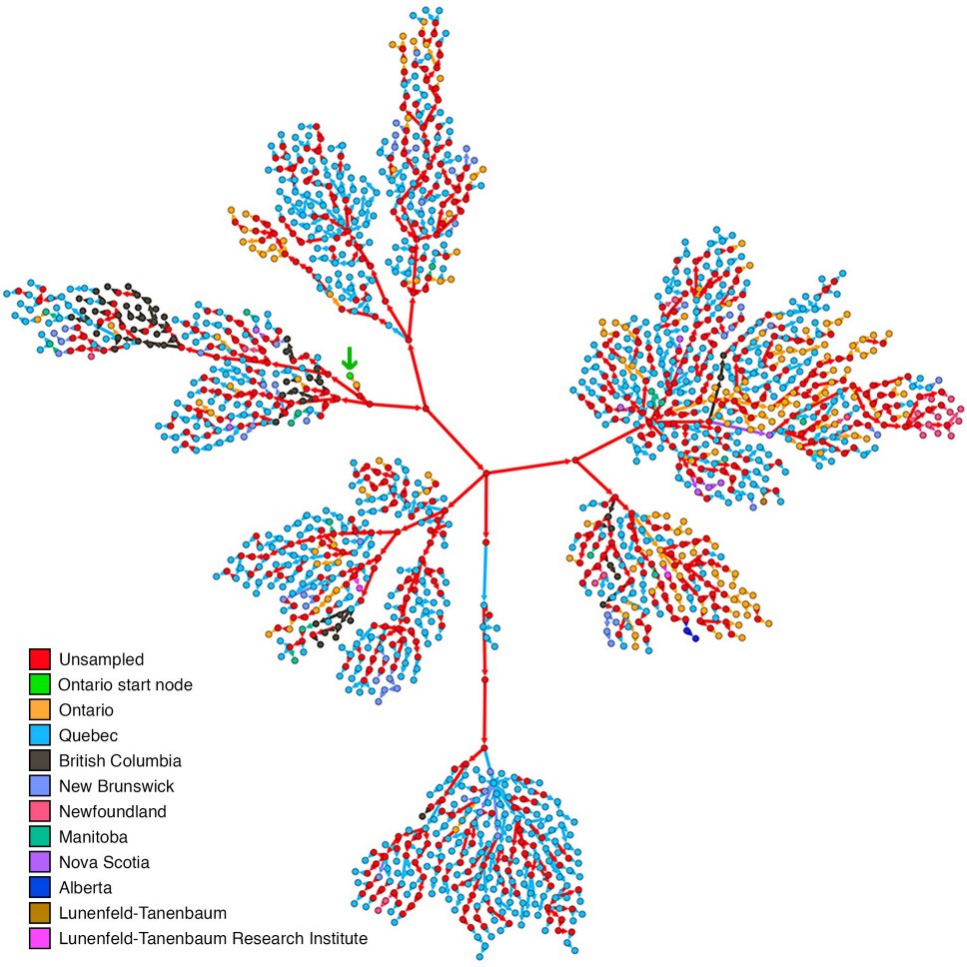
The transmission diagram obtained for the Canada dataset. The transmission diagram was colour coded based on the regions from which the sampled nodes were generated. The first node of infection, originated in Ontario, has been marked by a green arrow. The inferred unsampled sources of infection are coloured in red and the colour of the arrow depicts the recipient node. The diagram clearly depicts the effect of physical distancing with clear isolation of the nodes of the same colour. The unsampled nodes (35.63% shown in red) make up the rest of the transmission chain.

The first reported case of COVID-19 infection in Canada was seen in January of 2020 in Ontario, Toronto followed by reports of documented infections in several regions of British Columbia by early February followed by cases in Quebec and finally to the rest of Canada by late March. During this time period, a more extensive sampling of infected individuals was carried out [42–44]. The transmission network diagram produced by the pipeline (Fig 2) successfully inferred the transmission events consistent with the observations in these reports. In alignment with literature the pipeline inferred the first incidence of infection to have occurred in Ontario in January of 2020. From the total number of 1 496 nodes present in the transmission network, 963 of them were sampled nodes and the remaining 533 nodes which accounted for 35.63% of the network were inferred as unsampled sources. These inferences are consistent with the extensive testing deployed across Canada during that period and estimations of undiagnosed individuals [43]. There was a marked increase in the testing rate for COVID-19 in Canada beginning from early March. By late March the Canadian diagnostic laboratories were performing over 12 000 tests per week with an average of over 1 000 positive tests [45]. As such this accrual of testing combined with the quantity of cases arising at this time is consistent with our findings.

The transmission diagrams show the clustering of nodes within the same region as regions started to slow the spread of SARS-CoV2 infection consistent with the promotion of physical distancing practices in Canada by mid-March [46]. This observation would support the success of infection prevention approaches such as the “Atlantic Bubble” (ie. the Provinces of New Brunswick, Prince Edward Island, Nova Scotia, Newfoundland and Labrador) established on the 3^rd^ of July 2020 [47]. This success appeared evident when examining the transmission of COVID-19 in Newfoundland (which belongs to the “Atlantic Bubble”). Based on the sequences used, the phylogenetic pipeline inferred no in-province transmission events for Newfoundland from March of 2020 to July 2020 which could lend support to the reported success of the Atlantic Bubble. We believe, the transmission diagram obtained from the phylogenetic pipeline, could potentially be used to examine and easily visualize the effects of various infection control protocols.

### The analysis of the spread of SARS-Cov-2 in Russia

**Fig 3:**
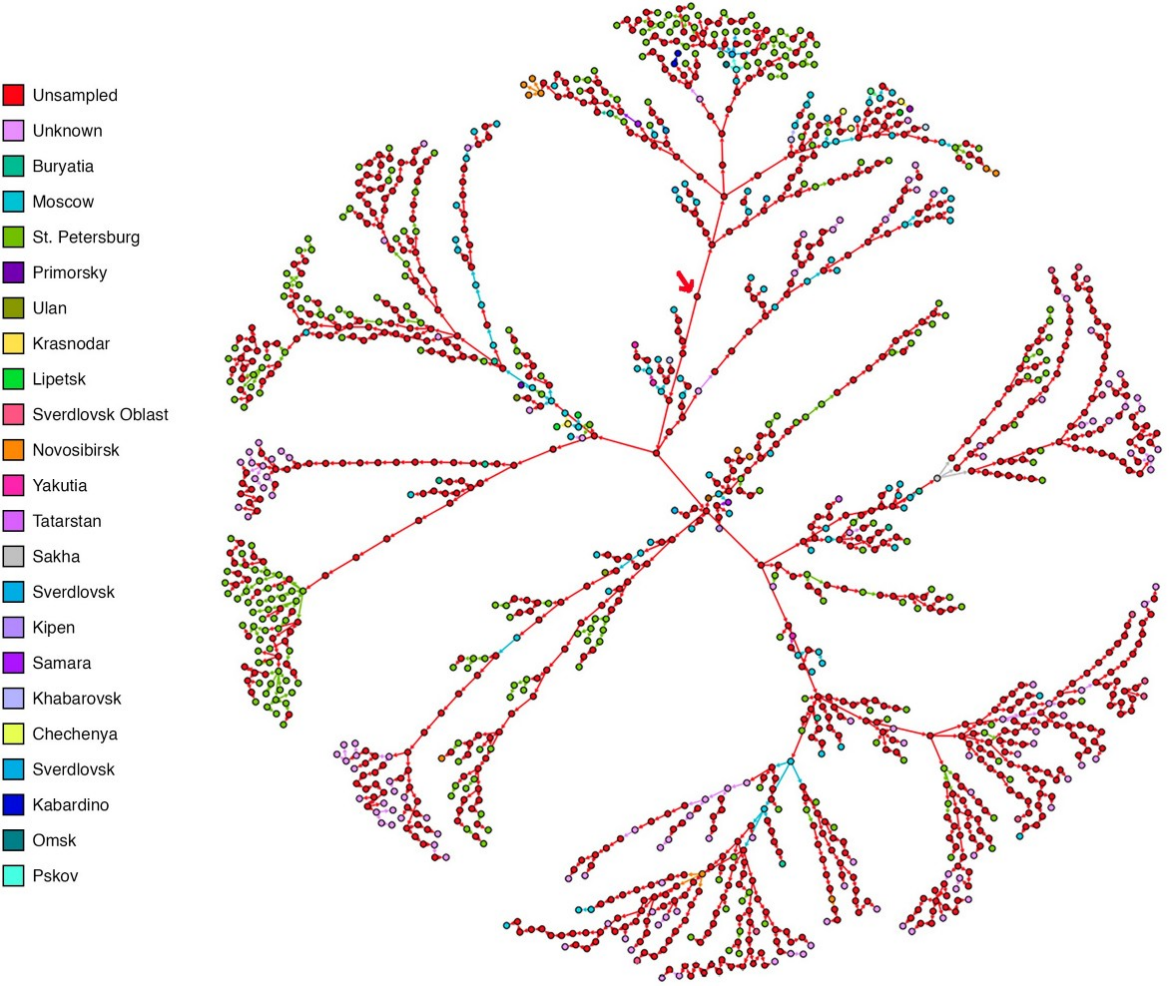
The transmission network obtained from the Russian data set. The transmission network inferred considerably large number of unsampled sources (61.06% shown in red) in contrast to the sampled sources. We can see clustering of the nodes in areas as such as St. Petersburg (in green) from which a majority of the sampled sequences are present (19.04%). However, this level of granularity was difficult to obtain for the other regions of Russia due to the lack of sequences from these regions. In spite of this shortcoming due to the pipelines robustness we are able to better understand the transmission by the filling in the potential links through the inference of unsampled sources of infection, suggesting a highly dynamic spread of the virus through the population with no single source. Despite this it was still able to infer transmission events that were authenticated by the available literature. The “source” of infection in this network is an inferred unsampled node marked by a red arrow (almost center in the transmission diagram).

The transmission diagram of the Russian dataset (Fig 3) appears consistent with the literature, despite the absence of extensive epidemiological data. TransPhylo inferred 61.06% as non-sampled sources of infection, consistent with the apparent lower sampling. Kozlovskaya et al. [48] state that the first COVID-19 cases identified in Russia were found in Moscow, and that national transmission may have started from there, and would have been bolstered by new arrivals to other cities. This is backed up by the presence of Moscow-derived samples appearing at a large portion of larger cluster junctions in our transmission diagram (Fig 3), indicating travel may have led to spread and clusters in cities such as Saint Petersburg. Additionally, Komissarov et al. [49] have identified nine distinct transmission networks within the Russian Federation, making use of similar data obtained from GISAID [49]. Several of the transmission networks deduced by Komissarov et al. were transmissions from Moscow to Yakutia, from Krasnodar to Orenburg, and from Moscow to Sverdlovsk. The intermingling of various regions in our generated transmission pathways are consistent with this dynamic spread reported for the Russian Federation (Fig 3). TransPhylo did also show transmission between many regions and transmission through various inferred unsampled sources which could potentially tie the transmission networks identified Komissarov together [49]. Additionally, several larger transmission clusters were reported in Saint Petersburg, which are recapitulated in our analyses. These patterns appear to coincide with outbreaks at the Vreden Hospital which occurred somewhere from late-March to early-April [49].

### Analyses of the spread of SARS-CoV-2 in New York State and the inference of cross-species transmission

**Fig 4:**
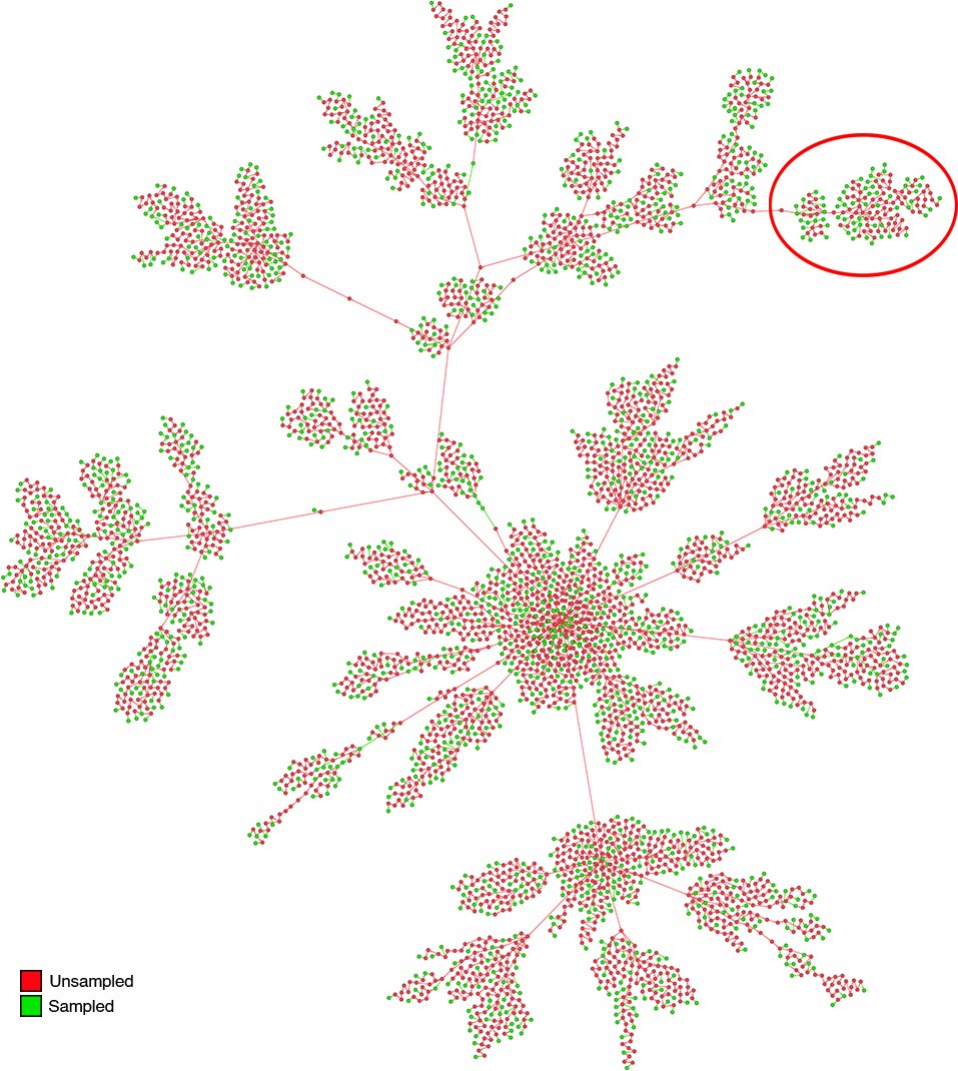
Transmission network diagram obtained from the New York State dataset. The number of inferred unsampled sources (65.73% shown in red) greatly outnumber the sampled sources (34.27% shown in green). The clear formation of a central cluster of nodes can be seen due to a high number of introductions of the infection into the region from the outside. The Bronx Zoo area is circled and further shown in Fig 5.

The New York State dataset further tested the capabilities of the proposed pipeline. The resulting transmission diagram (Fig 4) appears to have an epicenter with a large number of unsampled nodes overtaking the number of sampled cases. The central node’s inferred infection date of December 2019 coincides with the world’s first reported cases of COVID-19 [50]. Due to the large numbers of inferred unsampled sources of infection comprising of over 65.73% of the network coupled with the lack of clustering of nodes from the same region or location, it appears that on average, in New York State there were potentially various introductions through travel and broad range of clusters of infection, and the available physical distancing practices did not appear to limit the spread. The available literature on the spread of SARS-CoV-2 in New York State seems to be consistent with this notion [51–53].

**Fig 5:**
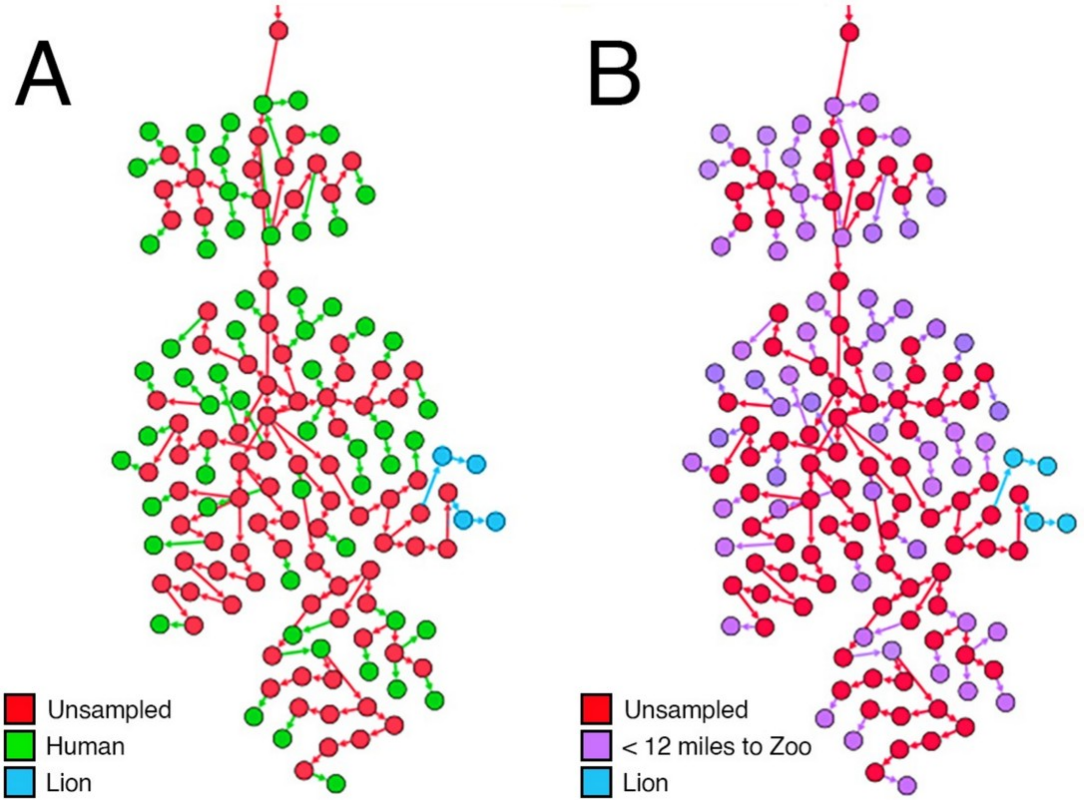
The inference of the cross-species human to lion transmission of the SARS-CoV-2 virus. **(A)** Colour coded based on the host organism. Based on this analysis the virus transmitted from a cluster of human hosts (green) to the lions (blue). Two lions have been initially infected from the human hosts and they, in turn, have infected the other lions. **(B)** Colour coded based on the vicinity of the sampled region from the Bronx Zoo, New York City. The entire cluster in that region is from areas that are less than 12 miles from the zoo.

The phylogenetic pipeline was also able to infer SARS-CoV-2 transmission from human to animal. In April of 2020 seven tigers and four lions at the Bronx Zoo, New York City were infected with SARS-CoV-2. The subsequent extensive analysis showed that the cross-species transmissions were from a human to lion and human to tiger [18]. Our pipeline was capable of making this inference from the genetic data available (Fig 5A). The pipeline was also inferring a series of unsampled sources of infection that arose from sampled sources and linked to the infection of the lions at the Bronx Zoo. The analysis also suggested that the subsequent *Panthera sp*. infections were the result of animal to animal infections and not solely human to animal transmission. To confirm the plausibility of this inference we analysed the epidemiological data of the nodes surrounding the lion and tiger data points (Fig 5B). This revealed that these sampled sources were obtained at laboratories (including the New York University Medical Center and QDX Pathology) less than 12 miles from the Bronx Zoo. Assuming that all sources sampled at these sites lived in the area serviced by these laboratories, this information reinforced the robustness of the pipeline.

## Discussion

Our analysis clearly indicated that after finetuning the combination of Bayesian phylogenetics and TransPhylo results in a pipeline able to infer transmission networks and including the inference of unsampled sources of infection. This pipeline offers a significant addition to current infectious diseases modeling approaches and gives valuable insights into the transmission dynamics in a population, and the effect of public health interventions.

Through a process of repeated iterations and validation, we were able to fine-tune the five-step phylogenetic approach. Using our previous work on HIV as the foundation of the pipeline we meticulously calibrated the protocol to capture the dynamics of causative SARS-CoV-2 virus and the COVID-19 pandemic. The “parameter optimised” pipeline inferred the key transmission events using genomic data without apparent extensive contact tracing. The main inconsistencies we identified in the pipeline were observed in BEAST2 inferences of the “become uninfectious rate” and “reproductive number”. Theses inconsistencies are most likely caused by the comparatively small sample size and the limited sample available and the scarcity of data for the period assessed. Normally one uses data spanning many years for such work, but this was unavailable at the start of the COVID-19 pandemic. As more and more jurisdictions are including routine and large-scale viral genomic data collection as part of their COVID-19/SARS-CoV-2 surveillance, the lack of sampling will become less of an issue. Thus, the inference of reproductive rates and analyzing the effects of public health would become more accurate. Regardless, our analyses showed that even with the limited datasets, the final output being the inference of transmission networks in a population appeared consistent with the literature.

The ability to infer the presence of unsampled sources of infection leads to a number of interesting possibilities. Through the optimization of TransPhylo for COVID-19, we have been able to explore some of these in the context of zoonoses as can be seen in the New York State dataset. TransPhylo works on the assumptions that the pandemic is ongoing and future transmissions continue to occur even after the completion of sample collection. Using a Bayesian approach with timed trees, we took great lengths to ensure that our pipeline produces the most statistically sound tree. Together, with these assumptions and the optimization of the tools’ sampling and infectious rates according to the region of study, we were able to infer transmission networks consistent with the literature.

In all of our tests, the pipeline was able to infer the transmission of SARS-CoV-2 and gave a rather clear perspective on the pandemic’s progression. The pipeline outcomes and inferences matched the published literature. In the analysis of the Russian and Canadian datasets, we observed the pipeline accurately inferred the evolution of the pandemic. We believe we were also able in our transmission diagrams to see the effect of the countermeasures applied by public health authorities on the pandemic. For instance, in the Canadian context the Atlantic Bubble (i.e. the Atlantic Provinces of Canada has been mentioned a lot as demonstration how travel restrictions could limit viral spread. Our analyses based on the data from GISAID, would support this notion. We observed limited spread within the Atlantic Bubble, and all introductions appeared to be linked other regions in Canada. It needs to be noted the sequence data from the Canadian Atlantic Provinces was not extensive enough, so we cannot exclude that transmission actually occurred at the local level not caught by the limited sequences available.

The significant power of inferring unsampled sources of infection to complete a transmission network was probably most apparent in handling “problematic” data sets such as New York State. At the time of data collection, New York State was a COVID-19 epicenter where the rate of infection had clearly surpassed the sequence sampling rate. The population was apparently highly mobile with people entering the state as well as local travel [51, 53]. The transmission network diagram was able to infer this mobile population and connect the scattered nodes from different regions in New York through the inference of unsampled sources of infection. The pipeline was able to use partial genomic and epidemiological data to paint a rather comprehensive picture of the pandemic’s status in the state, suggesting many different smaller as well as larger transmission chains linking various geographic regions in the state. We expanded this step further to infer the inter-species transmission of the virus from humans to lions. The inclusion of viral sequences obtained from the lions at the Bronx Zoo resulted in the generation of a transmission tree that depicted the lions being infected from human sources. This was consistent the original reports [18]. The validity of the result was further confirmed by the surrounding nodes of sampled sources being from the neighboring areas surrounding the Bronx Zoo. This cross-species transmission had intrigued health care workers and researchers until it was validated by extensive laboratory testing [18]. The pipeline was able infer this event using the available publicly available genomic data. Moreover, for the Bronx Zoo case it is of interest that the pipeline also inferred animal to animal transmissions, rather than only human to animal transmissions. It may therefore be of interest to apply these approaches further to the data obtained from the various mink farm outbreaks to examine the extend of human to animal, animal to animal, and animal to human transmissions in more detail [54].

We recognize that the “Unsampled” individuals in our analysis, may have been tested and have been found positive for SARS-CoV-2 but these sources were not sequenced. In regions without extensive sequencing practices or infrastructure this is a common hurdle; and it will not be feasible to sequence their entire symptomatic infectious population. It is in such environments that our solution serves the highest practicality. By combining the unsampled numbers inferred by our pipeline with the number of SARS-CoV-2 positive test numbers in the population, it could give insight into the number of undiagnosed asymptomatic and pre-symptomatic individuals in the population. Thereby providing the governing body with a more detailed overview of the ongoing epidemic.

Our analyses is limited by the fact that we used public available data, and as such no comprehensively sequenced samples are available. This of course, could result in an underestimation of the inference of unsampled individuals. However, despite this limitation the overall picture emerging from the inferences were consistent with the literature. With more and more sequencing data being available for different regions, the accuracy of inferring the correct number of undiagnosed and asymptomatic individuals will increase. This while using anonymous data and without intensive contact tracing. The latter will again help improve epidemiological modeling and interpreting the effects of public health interventions on infection control and spread.

## Conclusion

We were able to reconstruct the transmission patterns in a population and establish a clear picture of an infected population despite incomplete sampling. With the refinements we applied to the pipeline we demonstrated the potential utility of these tools for COVID-19. We also believe that these types of robust pipelines can truly be front-line tools in the battle against infectious diseases beyond SARS-CoV-2.

## Supporting information

S1 Fig The trace diagram for the New York dataset after the combination of all the BEAST2 log files. As depicted in the figure all parameters have an ESS score above 200.
(TIF)

S2 Fig MCMC trace diagrams. Representative MCMC trace diagrams generated by TransPhylo for the optimization of its four variable parameters.
(TIF)

S1 Table GISAID accession numbers of sequences used. (XLSX)
