# Supplementary figures and images for: Reconstructing SARS-CoV-2 infection dynamics through the phylogenetic inference of unsampled sources of infection
Source: PLoS One. 2021 Dec 15;16(12):e0261422. doi: 10.1371/journal.pone.0261422 (PMC8673622; doi:10.1371/journal.pone.0261422)

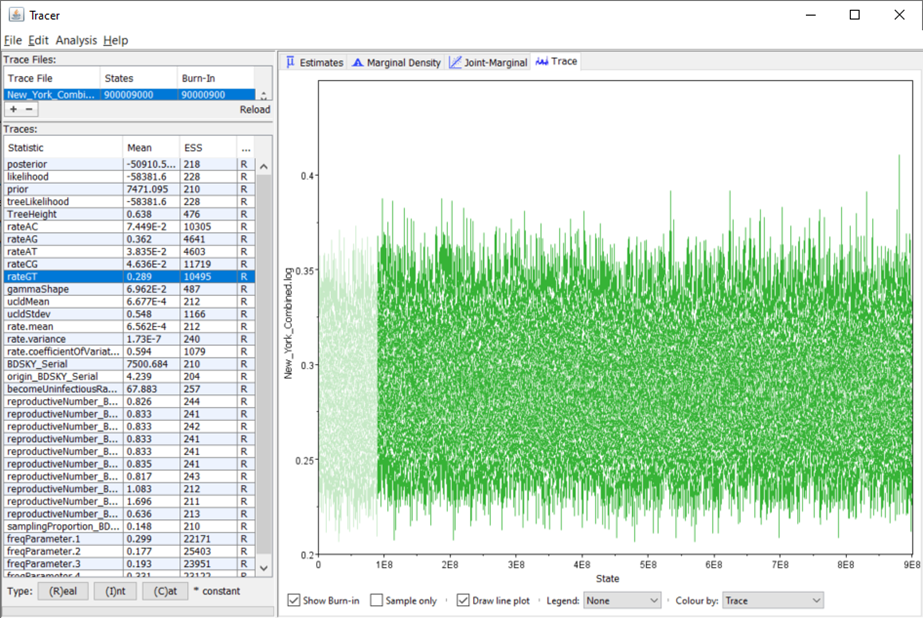

Supplement: S1 Fig — As depicted in the figure all parameters have an ESS score above 200. (TIF) [file pone.0261422.s001.tif]

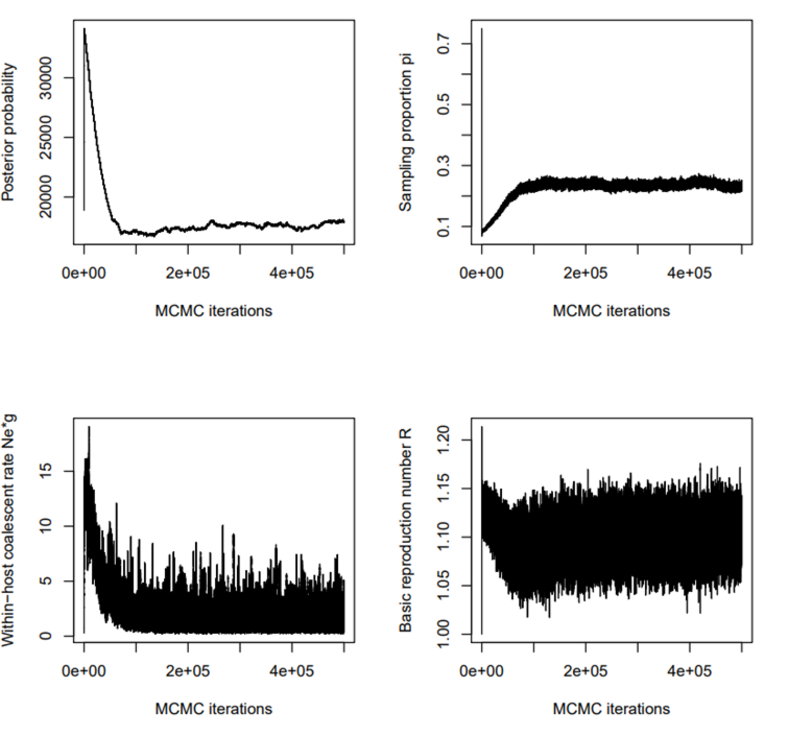

Supplement: S2 Fig — Representative MCMC trace diagrams generated by TransPhylo for the optimization of its four variable parameters. (TIF) [file pone.0261422.s002.tif]
